# Regression and Eradication of Triple-Negative Breast Carcinoma in 4T1 Mouse Model by Combination Immunotherapies

**DOI:** 10.3390/cancers15082366

**Published:** 2023-04-19

**Authors:** Saifun Nahar, Yue Huang, Bethany A. Nagy, John A. Zebala, Dean Y. Maeda, Udo Rudloff, Joost J. Oppenheim, De Yang

**Affiliations:** 1 Cancer and Inflammation Program, Center for Cancer Research, National Cancer Institute at Frederick, Frederick, MD 21702, USAoppenheij@mail.nih.gov (J.J.O.); 2 Rare Tumor Initiative, Pediatric Oncology Branch, Center for Cancer Research, National Cancer Institute, Bethesda, MD 20892, USA; 3 Syntrix Pharmaceuticals, Auburn, WA 98001, USA

**Keywords:** TNCB, 4T1, ICB, HMGN1, SX682, R848, FSL-1, immunotherapy, alarmin, cancer therapy

## Abstract

**Simple Summary:**

There is currently no effective therapy available for triple-negative breast cancer. To look for potentially effective treatment, we used the 4T1 mouse model of triple-negative breast carcinoma to study the therapeutic response of TheraVac (an antitumor therapeutic vaccination regimen) in combination with FSL-1 and/or SX682. The data show that 4T1 tumors can be successfully treated with two TheraVac modifications, with the development of anti-4T1 immune responses in the treated mice. Therefore, these TheraVac modifications have potential to be developed into effective immunotherapies for triple-negative breast cancer.

**Abstract:**

Triple-negative breast carcinoma (TNBC) is one of the most aggressive types of solid-organ cancers. While immune checkpoint blockade (ICB) therapy has significantly improved outcomes in certain types of solid-organ cancers, patients with immunologically cold TNBC are afforded only a modest gain in survival by the addition of ICB to systemic chemotherapy. Thus, it is urgently needed to develop novel effective therapeutic approaches for TNBC. Utilizing the 4T1 murine model of TNBC, we developed a novel combination immunotherapeutic regimen consisting of intratumoral delivery of high-mobility group nucleosome binding protein 1 (HMGN1), TLR2/6 ligand fibroblast-stimulating lipopeptide (FSL-1), TLR7/8 agonist (R848/resiquimod), and CTLA-4 blockade. We also investigated the effect of adding SX682, a small-molecule inhibitor of CXCR1/2 known to reduce MDSC trafficking to tumor microenvironment, to our therapeutic approach. 4T1-bearing mice responded with significant tumor regression and tumor elimination to our therapeutic combination regimen. Mice with complete tumor regressions did not recur and became long-term survivors. Treatment with HMGN1, FSL-1, R848, and anti-CTLA4 antibody increased the number of infiltrating CD4+ and CD8+ effector/memory T cells in both tumors and draining lymph nodes and triggered the generation of 4T1-specific cytotoxic T lymphocytes (CTLs) in the draining lymph nodes. Thus, we developed a potentially curative immunotherapeutic regimen consisting of HMGN1, FSL-1, R848, plus a checkpoint inhibitor for TNBC, which does not rely on the administration of chemotherapy, radiation, or exogenous tumor-associated antigen(s).

## 1. Introduction

Modulation of patients’ own immune system is a promising strategy for the treatment of patients afflicted with cancer. Recent advances in immunotherapy have transformed the care and favorably changed the outcomes for many cancer patients. Immunotherapy approaches in the form of checkpoint inhibitor monoclonal antibody (CIMA) or chimeric antigen receptor (CAR) T-cell therapy have become first- or second-line treatment options, and in some patients conferred sustained, durable treatment responses generally not observed with standard systemic chemotherapy. To date, these positive findings are limited to immunologically “hot” cancers. To the contrary, for most patients with solid-organ cancers, which are classified as immunologically “cold”, the promise of immunotherapy via T-cell activation has not materialized in improved clinical outcome. These tumors create an immune milieu which excludes cytotoxic T cells and/or induces “exhausted” T-cell phenotypes through an abundance of immune-evasive cues frequently involving tumor-educated, dysfunctional myeloid immune cell populations. With multiple approaches including the adoptive transfer of either tumor-infiltrating T lymphocytes (TIL) or CAR-T cells, cytokine therapy (interferons, IL-2, IL-12), cancer vaccines, or therapies targeting immune checkpoint (such as antibodies to LAG-3, CD40, TIGIT, or TIM-3) are under active investigation; combination strategies which harness antitumor cues of multiple components of the immune system and which target mechanisms of adaptive resistance early have started to emerge as the most promising strategies [1,2,3,4,5,6,7]. Combination therapies are designed to generate primary, possibly synergistic, immune responses by reducing or recalibrating immunosuppressive components present of tumor microenvironment (TME)-like regulatory T cells (Tregs), myeloid-derived suppressor cells (MDSCs), tumor-infiltrating dendritic cells (TiDCs), or tumor-associated macrophages (TAM). Therapeutically, the reduction of the immune suppressive milieu of the TME aims to halt/delay the development of adaptive resistance after ICB therapy, which is characterized by a lack of T-cell responses due to re-exhaustion of T effector cells, upregulation of other immunosuppressive signaling receptors, disruption of antigen presentation, and/or the evolution of interferon resistance. The TME of solid-organ cancers is infiltrated with various types of immune cells, including innate immune cells like dendritic cells (DCs), natural killer cells, MDSCs, or TAMs, as well as adaptive immune cells, including effector T cells (CD8+ and CD4+ T cells), or regulatory T cells (Tregs) [8]. DCs work as professional antigen-presenting cells (APCs) by processing and presenting antigen and conveying stimulatory signals to T cells. In addition, DCs also activate NK cells and B cells [9]. However, tumor cells present in the TME paralyze tumor-infiltrating DCs (TiDCs), rendering them immature with elevated expression of inhibitory molecules and lowered antigen-presenting capacity. Cancer cells usurp TiDC function for pro-survival gains, as paralyzed/immature TiDCs carry immunosuppressive rather than immunostimulatory properties and contribute to immune evasion and loss of tumor control [10]. Hence, recalibrating TiDCs and activating TiDC function via reprogramming towards an antitumor phenotype is a desirable strategy to enhance the efficacy of cancer immunotherapy.

Toll-like receptor (TLRs) activation is the most often used path to induce activation and maturation of dendritic cells [11]. Our laboratory has previously shown that HMGN1, a TLR4 ligand, has the capacity to activate DCs and promote Th1 immune responses [12] and, in combination with a TLR7/8 agonist, resiquimod, synergistically activates DCs to produce proinflammatory cytokines [13]. In the following work, our group showed that the generation of effective antitumor immunity against a variety of preclinical solid-organ cancer models including CT26 colon cancers, RENCA renal cell carcinoma, EG7 thymoma, and Lewis lung carcinoma is improved when combining HMGN1, R848, and checkpoint inhibitor blocking antibody, a strategy which was termed TheraVac [14,15]. However, preclinical models of breast cancer were shown to be least sensitive with limited response rates observed to TheraVac.

Here, we aimed to investigate the therapeutic impact of an improved TheraVac regimen on TNBC using the syngeneic 4T1 murine breast cancer model. Like observations in patients with TNBC, the murine carcinoma 4T1 model is refractory to checkpoint blockade monotherapy, indicating the need for the incorporation of additional immuno-oncology targets to yield effective combinational therapies in this disease [16,17,18,19]. In this study, ICB therapy was initially given together with N1 and R848 to activate TiDCs and promote the generation of antitumor immune responses. To improve the current regimen, we extended the TheraVac approach by adding the TLR2/6 ligand fibroblast-stimulating lipopeptide 1 (FSL-1), which we combined with HMGN1 and/or R848 and ICB. Furthermore, SX682, a small-molecule inhibitor of the chemokine receptors CXCR1/2 capable of blocking their binding with IL8 and hence reducing the trafficking of MDSCs towards 4T1 tumors [20,21], was also incorporated into the therapeutic approach. Our data demonstrated that combinational therapies including modified TheraVac (TheraVac^M^, consisting of N1, FSL-1, and anti-CTLA4), TheraVac plus FSL-1 (TheraVac^Plus^, consisting of N1, R848, anti-CTLA4, and FSL-1), and TheraVac^Plus^, and SX-682 (TheraVac+ and SX-682, consisting of N1, R848, FSL-1, anti-CTLA4, and SX682) caused significant regressions of established 4T1 tumors as well as reduction of metastasis burden to the lungs. TheraVac^Plus^ and TheraVac^Plus^ + SX682 therapies successfully eradicated the established 4T1 tumors after 5 to 6 rounds of treatment and the resultant tumor-free mice became long-term survivors. Therefore, combination therapies of extended immunostimulant combinations with ICB appear to be a promising cancer treatment strategy for TNBC.

## 2. Materials and Methods

### 2.1. Mouse and Cell Line

Balb/c mice (8–12-week-old, female) were obtained from the Jackson laboratory. NCI-Frederick is accredited by AAALAC International and follows the Public Health Service Policy for the Care and Use of Laboratory Animals. Animal care was provided in accordance with the procedures outlined in the “Guide for Care and Use of Laboratory Press” (Washington, DC, USA). All animal studies were approved by the Institutional Animal Care and Use Committee (IACUC) of the National Cancer Institute at Frederick (Frederick, MD, USA).

Mouse 4T1 breast cancer cell line (CRL-2539) used in the present study was purchased from the American Type Culture Collection (ATCC, Manassas, VA, USA). 4T1 cells were regularly examined for contaminants with the Molecular Testing of Biological Materials (MTBM) test (Animal Health Diagnostic Laboratory, NCI-Frederick, Frederick, MD, USA). The morphology, in vitro and in vivo growth rate, and metastatic ability of cell lines were routinely monitored. 4T1 cell line was cultured in DMEM (Corning, Tewksbury, MA, USA) supplemented with 10% FBS (Gemini, West Sacramento, CA, USA), 2 mM glutamine, 25 mM HEPES buffer (Quality Biologicals, Gaithersburg, MD, USA), 100 μg/mL penicillin, 100 μg/mL streptomycin, and 50 μM 2-mercaptoethanol at 37 °C in a humidified incubator with 5% CO_2_.

### 2.2. Generation and Treatment of DCs

Human monocyte-derived DCs (MoDCs) and mouse bone marrow-derived DCs (BMDCs) were generated, as described previously [13]. In brief, bone marrow progenitors were isolated from femur and tibia and incubated at 0.5~1 × 10^6^ cells/mL in complete RPMI 1640 medium (Corning) containing 10% FBS (Gemini), 2 mM glutamine (LONGA), 25 mM HEPES, 100 μg/mL penicillin (Corning), 100 μg/mL streptomycin (Corning), and 50 μM 2-mercaptoethanol and 20 ng/mL of murine GM-CSF (PeproTech, Rocky Hill, NJ, USA) at 37 °C in a humidified incubator with 5% CO_2_ for 6 days with medium change on the 2nd and 4th days of culture to generate immature DCs. To generate human monocyte-derived (MoDCs), peripheral blood monocytes from healthy volunteers were isolated from peripheral blood PBMCs by MACS using a human CD14 isolation kit (Miltenyi Biotec, Gaithersburg, MD, USA) and incubated in the presence of 50 ng/mL of human GM-CSF (PeproTech) and human IL-4 (PeproTech) for 5 days, as previously described. Immature BMDCs or MoDCs were incubated with fresh medium only or fresh medium containing the combination of N1 (250 ng/mL), FSL-1 (0.5 ng/mL), and R848 (250 ng/mL). After 48 h, treated DCs were collected for analysis of phenotypic markers. Supernatants were used for measuring the concentration of cytokines using V-PLEX ELISA (TNF-α and IL-12p70) (Meso Scale Discovery, Rockville, MD, USA).

### 2.3. Establishment of Mouse Tumor Models and Treatment

Female (Balb/c, n = 5–10, 8–12-wk-old) were shaved and subcutaneously injected with 0.1 mL PBS containing 4T1 (2 × 10^6^/mL) cells into the right flank. The appearance and size of tumors as well as mouse body weight were measured twice a week. The length and width of tumors were measured by a caliper. Tumor size was calculated by the formula: (L × W2)/2. When tumors became palpable, tumor-bearing mice were randomized to the respective treatment arms. SX682-medicated chow (200 mg/kg body weight/day; Research Diets obtained under a Cooperative Research and Development Agreement with Syntrix Pharmaceuticals) was started in the respective group, whereas non-medicated chow was given to the rest of the control arm. When tumors reached 5 to 7 mm in any diameter, tumor-bearing mice were treated with intraperitoneal (i.p.) injection of anti-CTLA4 (200 μg/0.2 mL/mouse), intratumoral (i.t.) injection of N1 (10 μg/0.05 mL/mouse), R848 (10 μg/0.05 mL/mouse), and FSL-1 (5 μg/0.05 mL/mouse) or PBS. Anti-mouse CTLA-4 (CD152) (clone 9H10) were purchased from Bio X Cell (Lebanon, NH, USA). FSL-1 and R848 were purchased from Invivogen (San Diego, CA, USA). The GMP level of recombinant N1 was produced in-house. To determine metastasis, lungs retrieved from the mice that reached end point were soaked in Bouin solution for three days before the metastasis nodules on the surface of the lung were enumerated.

### 2.4. Dissociation of Tumors and Draining Lymph Nodes

Following treatments, residual tumors and tumor-draining lymph nodes were harvested and processed in the laboratory to make single-cell suspensions. Tumors cut into approximately 1 mm^3^ cubes were digested in an enzymatic cocktail (at a ratio of tumor:cocktail = 1 g:25 mL) at 37 °C with constant slow mixing (in a rotator at 80 rpm) for 45 min. At the end of digestion, the tubes were put in a vertical position at room temperature for 5~10 min to allow the undigested tumor pieces to settle at the bottom of the tubes, and the upper phase of cell suspension was transferred to a fresh tube (on ice). A fresh cocktail prewarmed to 37 °C was added before the tubes were subjected to the 2nd round of digestion. The enzymatic cocktail was freshly prepared and contained 0.17 mg/mL of collagenase I (Worthington Biochemical Corp. Lakewood, NJ, USA), 0.056 mg/mL of collagenase II (Worthington Biochemical), 0.17 mg/mL of collagenase VI (Worthington Biochemical), 0.025 mg/mL of deoxyribonuclease I (Worthington Biochemical), and 0.025 mg/mL of elastase (Worthington Biochemical) in Leibovitz L-15 medium (Meditech), as described previously. The cell suspensions collected from both rounds were pooled, passed through a 70 µm filter, washed 3 times with PBS, counted, and used single-tumor cell suspensions. Draining lymph nodes were put inside a 70 µm strainer that was placed in a dish containing ice-cold PBS, squeezed gently with pestle, and cell suspension outside of the strainer was collected, washed, counted, and used as single-lymph node cell suspension(s).

### 2.5. Immunostaining and Flow Cytometry

For detecting DCs surface markers, BMDCs were washed in FACS buffer (PBS containing 2% FBS and 0.05% NaN_3_), blocked with FACS buffer containing 2% normal mouse serum on ice for 10 min, and stained with a combination of FITC-anti-mouse I-A/I-E (BioLegend, San Diego, CA, USA, clone M5/11.4.15.2), Pacific blue-anti-mouse CD80 (BioLegend, Clone 16-10A1), PE-anti-mouse CD83 (Biolegend, clone Michel-19), Percp/Cyanine 5.5 anti-mouse CD86 (Biolegend, Clone GL-1), and APC anti-mouse CD11c (BioLegend, clone N418) on ice for 20–30 min. MoDCs were washed in FACS buffer (PBS containing 2% FBS and 0.05% NaN_3_), blocked with FACS buffer containing 2% human AB serum on ice for 10 min, and stained with a combination of FITC mouse anti-human CD80 (BD Pharmingen^TM^, San Jose, CA, USA, clone L307.4), APC mouse anti-human CD83 (BD Pharmingen^TM^, clone HB15e), PE mouse anti-human CD86 (BD Pharmingen^TM^, clone 2331 FUN-1), Pacific Blue™ anti-human HLA-DR Antibody (BioLegend, clone L243), PerCP/Cyanine5.5 anti-human CD11c Antibody (BioLegend, clone3.9). Stained DC samples were washed once with FACS buffer and three times with PBS, suspended in PBS, and analyzed using a BD LSRII SORP multichannel cytometer. For detecting infiltration of various subsets of leucocytes, MDSCs, effector/memory T cells in the tumor tissue and draining lymph node, single-cell suspension from digested tumor tissue, and dLN were stained with Pacific Blue™ anti-mouse CD45 Antibody (BioLegend, clone 30-F11), PE anti-mouse/human CD11b Antibody (BioLegend, clone M1/70), APC anti-mouse CD11c (BioLegend, clone N418), PerCP-Cy™5.5 Rat Anti-Mouse CD45R/B220 (BD Pharmingen^TM^, clone RA3-6B2), APC/Cyanine7 anti-mouse CD8a Antibody (BioLegend, clone 53-6.7), FITC anti-mouse CD4 Antibody (BioLegend, Clone GK1.5), APC anti-mouse CD62L Antibody (BioLegend, clone MEL-14), PE anti-mouse/human CD44 Antibody(BioLegend, clone IM7), APC anti-mouse F4/80 Antibody (BioLegend, clone BM8), PerCP/Cyanine5.5 anti-mouse Ly6C Antibody (BioLegend, clone HK1.4), FITC anti-mouse Ly6G Antibody (BioLegend, clone 1A8), PerCP/Cyanine5.5 anti-mouse CD3 Antibody (BioLegend, clone 17A2) and stained samples were analyzed using a BD FACSymphony^TM^ A5 Cell Analyzer (Franklin Lakes, NJ, USA). All flow cytometry data were further analyzed using Flow Jo Software (10.8.1).

### 2.6. Cytotoxic T Lymphocyte (CTL) Detection by CD107a Mobilization Assay

A monolayer of 4T1 cells was prepared using 48-well plates by seeding 1 × 10^4^ cells/well and incubated at 37 °C in a humidified incubator with 5% CO_2_ overnight. The dLN cells were added to each well at a ratio of 50:1 (dLN:4T1). After 24 h of incubation, cells were harvested and stained with anti-CD8 efluor 450 and anti-CD107a-efluor660. Flow cytometry was conducted on a BD FACSymphony^TM^ A5 Cell Analyzer, and the final data were analyzed using Flow Jo software.

### 2.7. Statistical Analysis

The statistical significance among the multiple groups was analyzed by one-way ANOVA analysis with multiple comparison followed by Tukey’s post hoc test using GraphPad Prism version 9.2.0 (GraphPad, San Diego, CA, USA). The data were expressed as mean ± SEM of multiple experiments. Statistical significance was determined by * *p* < 0.05, ** *p* < 0.01, *** *p* < 0.005, and **** *p* < 0.001.

## 3. Results

**Figure 1:**
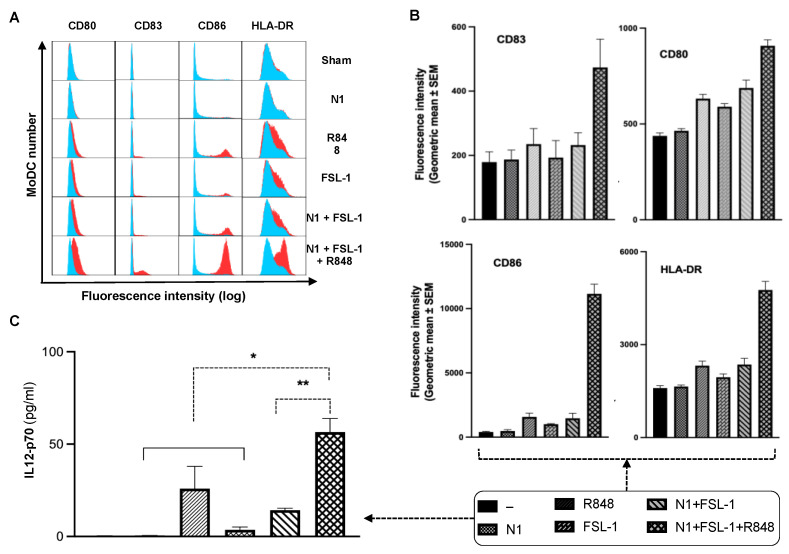
Synergistic activation of human monocytes-derived dendritic cells by HMGN1, R848, and FSL-1. (**A**) Overlay histograms (blue = sham/untreated DC, red = treated DCs) showing the cooperatively enhanced upregulation of DC surface costimulatory molecules (CD80, CD83, CD86) and MHC (HLA-DR) in response to combinational treatment with HMGN1+FSL-1+R848 (data of one experiment representative of three independent experiments). (**B**) Average mean fluorescence intensity of DC surface CD80, CD83, CD86, and HLA-DR (mean + SEM, N = 3). (**C**) Synergistic promotion of MoDC IL-12p70 production (* *p* < 0.05 and ** *p* < 0.001). Quantification of Multiplex ELISA measurements by Meso Scale Discovery (in pg/mL, mean + SEM, N = 3) of DC supernatant in response to combinational treatment.

Previously, our group developed a therapeutic vaccination regimen, a combination of N1, R848, and checkpoint inhibition (TheraVac), to treat various solid carcinomas in mouse models. TheraVac was delivered intratumorally to induce the maturation and activation of TiDCs for the induction of tumor-specific immunity. TheraVac significantly regressed, and eradicated, various cancers such as colon (CT26), kidney (RENCA), thymoma (EG7), lung (LLC) carcinomas in vivo [15], but failed to achieve similar efficacy in 4T1 breast carcinomas. Emerging evidence suggested that 4T1 breast carcinoma is comprised of a severely immunosuppressive microenvironment due to an abundance of MDSCs, which likely render TiDCs non-functional [22]. We hypothesized that the addition of effective immunostimulant(s) to the TheraVac regimen would be required for TheraVac to improve tumor control against TNBC. We selected FSL-1, which was shown to synergize with N1 to promote IL12 expression in DCs. To examine whether FSL-1, a TLR2/6 synthetic agonist, could cooperate with N1 and R848 to activate DCs, we treated human and mouse DCs with combinations of N1, FSL-1, and R848. Indeed, MoDCs treated with a combination of FSL-1, N1, and R848 showed higher levels of DC (Gating strategy shown in Supplementary Figure S1) surface expression of costimulatory molecules (CD80, CD83, CD86) and MHC class I (HLA-DR) (Figure 1A,B) and production of more IL12p70 (Figure 1C) compared to treatment with each reagent alone or a combination of N1 and FSL-1. Treatment with the combination of N1 and FSL-1 and R848 also more robustly activated BMDCs than treatment with N1 and FSL-1, as shown by upregulated expression of surface molecules (CD80, CD83, CD86), MHC class II (I-A/E) and enhanced production of IL12, and TNFα in BMDCs (Supplementary Figure S2A–C).

### 3.1. Effect of FSL-1 Combined with TheraVac (N1+R848+anti-CTLA4) on 4T1 Breast Cancer

**Figure 2:**
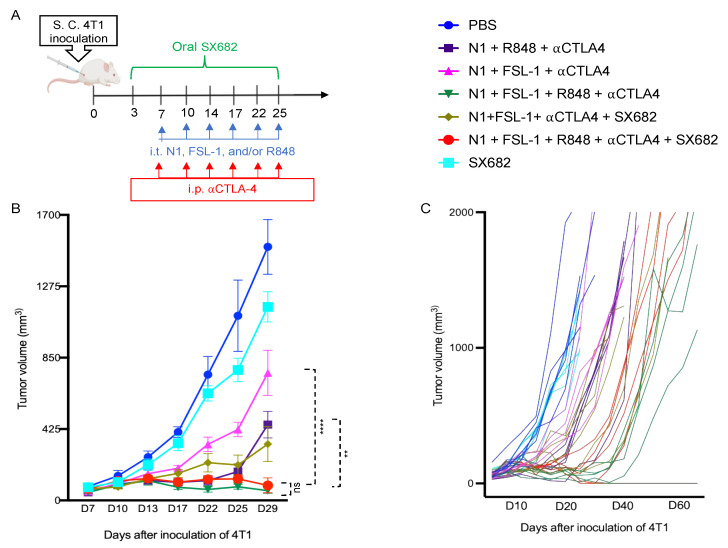

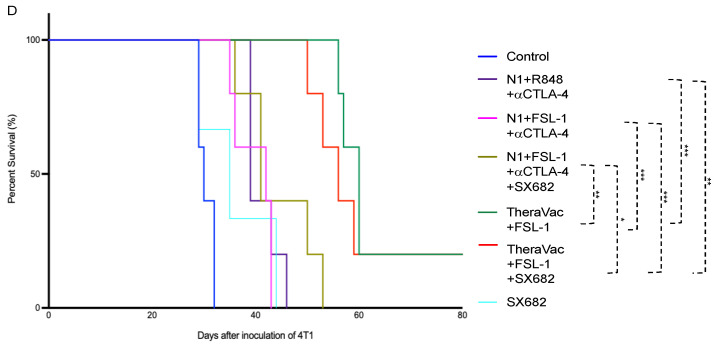
Antitumor effect of combination of HMGN1, FSL-1, and/or R848 with or without SX682 on mice bearing 4T1 breast carcinomas. (**A**) Scheme of 4T1 inoculation and treatment. (**B**) Effect on 4T1 tumor growth in mice (n = 5) being treated with Control/PBS (blue line), SX682 (aqua line), N1 + R848 + αCTLA-4 (purple line), N1 + FSL-1 + αCTLA-4 (pink line), N1 + FSL-1 +αCTLA-4 + SX682 (golden line), N1 + FSL-1 +αCTLA-4 + R848 (green line), N1 + FSL-1 +αCTLA-4 + R848 + SX682 (red line). Data are shown as mean ± SEM. (**C**) Effect of tumor growth in individual mouse of indicated groups. (**D**) The survival curves for each group are also indicated. * *p* < 0.05; ** *p* < 0.01; *** *p* < 0.001; and **** *p* < 0.0001.

Given the essential role of IL12 in Th1 polarization, which is critical for protective antitumor immunity, these data suggested that N1, FSL-1, and R848 potentially cooperate to trigger the generation of improved antitumor immune responses. Therefore, we incorporated FSL-1 into our TheraVac therapeutic regimen to determine whether anti-4T1 immunity is improved in vivo. 4T1-bearing mice were treated with various combinations in which N1, FSL-1, and R848 were administered intratumorally (i.t.), while anti-CTLA4 antibody was injected intraperitoneally into Balb/c mice bearing 4T1 tumors of approximately 0.5 to 0.7 cm in diameter. In line with previous work on the TheraVac approach, the i.t. route was selected as direct administration into the tumor tissue to induce the activation of TiDCs and to limit possible systemic toxicities [15]. In line with previous observations, treatment of 4T1-bearing mice with TheraVac consisting of N1, R848, and anti-CTLA4 significantly suppressed, but did not arrest, growth of 4T1 tumors. To test the cooperativity of FSL-1 with N1 and R848 in vivo, we first replaced the TLR7/8 agonist with FSL-1 in the formulation. Within a second, alternative approach, we also aimed to reduce the immunosuppression within the TME by inhibiting MDSC recruitment into 4T1 tumors using SX682, a small chemical antagonist capable of inhibiting IL8 interaction with its receptor CXCR1/2 [23,24]. Administered treatment schedules are summarized in Figure 2A, non-drug-infused chow or SX682-medicated chow was started on day 3 post 4T1 inoculation and continued until the last treatment. Treatment with TheraVac initially significantly inhibited the growth of 4T1 tumors prior to tumors escaping the administered immunostimulants and ICB (Figure 2B). CXCR1/2 receptor blocker monotherapy modestly delayed the growth of 4T1 tumors without achieving growth arrest (Figure 2B). Modified TheraVac consisting of N1+FSL-1+anti-CTLA4 (termed TheraVacM) in which R848 was replaced by FSL-1 yielded improved tumor control against 4T1 tumors (Figure 2B–D). The combination of TheraVacM and SX682 inhibited the growth of tumors more effectively than TheraVacM without SX682 (Figure 2B, ** *p* < 0.01). The combination of TheraVac^M^ with SX682 did not cure any of the 4T1-bearing mice. Next, we added R848 to N1, FSL-1, and anti-CTLA4 ICB. Among the tested combinations, TheraVacM plus SX682 and the combination of TheraVac and FSL-1 (termed TheraVacplus) showed the best therapeutic effect in the 4T1 model (Figure 2). Treatment with the combination of N1, FSL-1, R848, and anti-CTLA4 or the combination of N1, FSL-1, anti-CTLA4, and SX882 induced 4T1 tumor regressions with 20% of treated 4T1 tumor-bearing mice achieving a complete response and remaining in remission (Figure 2B–D). The overall survival of mice was also markedly prolonged, as tumor-free mice survived for more than 60 days from the day of inoculation (Figure 2D).

### 3.2. Effect of Combination of N1, R848, Anti-CTLA4, and FSL-1 (TheraVacplus) on 4T1 Metastasis to the Lungs

**Figure 3:**
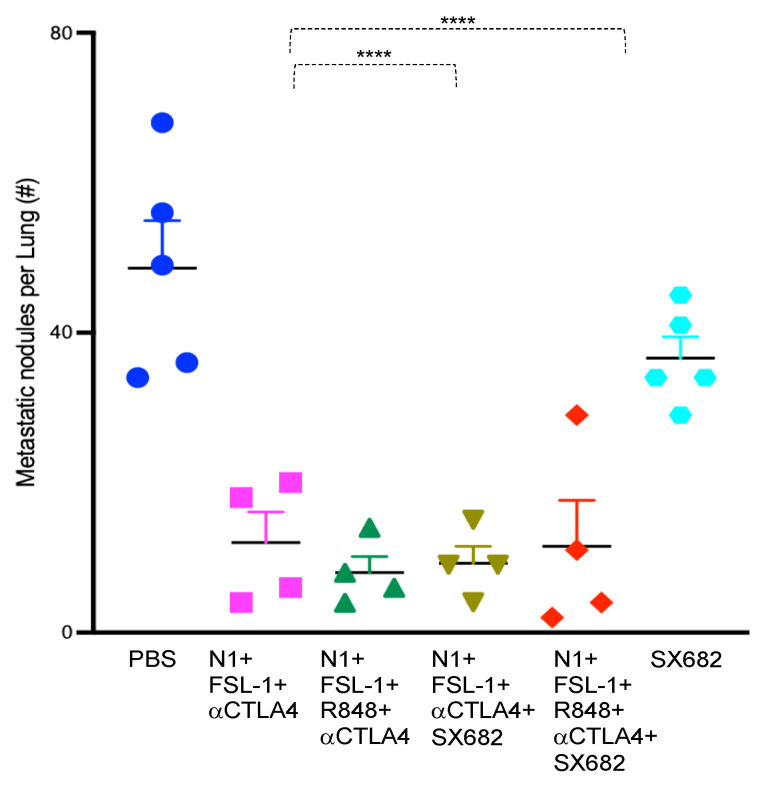
Inhibition of lung metastasis by combined therapeutic regimen. The number of metastatic nodules on the surface of the lung of mice in response to various treatments are shown as mean ± SEM (n = 4~5 mice). **** *p* < 0.0001.

Breast carcinoma-related death is primarily caused by metastasis [25]. To determine the impact of the TheraVac combinations on metastasis of subcutaneously implanted 4T1 tumors to the lung, we collected both lungs from tumor-bearing mice randomized to the different treatment regimens. We counted the number of metastatic nodules on the surfaces of all lobes of both lungs after soaking the lungs in Bouin solution for three days. The tested therapeutic regimens significantly reduced the number of metastatic lung nodules in comparison to the control. While control mice treated with vehicle had numerous large and small metastatic deposits macroscopically visible, mice randomized to the combination of N1, FSL-1, anti-CTLA4 (TheraVacM), TheraVacplus (N1, R848, anti-CTLA4, and FSL-1), TheraVacM and SX682, or TheraVacplus and SX682 had significantly lower numbers of surface nodules (Figure 3). The number of metastatic nodules was reduced by ~70% across all treated groups (Figure 3) with the exception of SX682-treated mice, where the reduction of the number of metastatic nodules was less.

### 3.3. Effect of TheraVacM and TheraVacplus on Immune Cell Infiltration in Tumor Tissues

**Figure 4:**
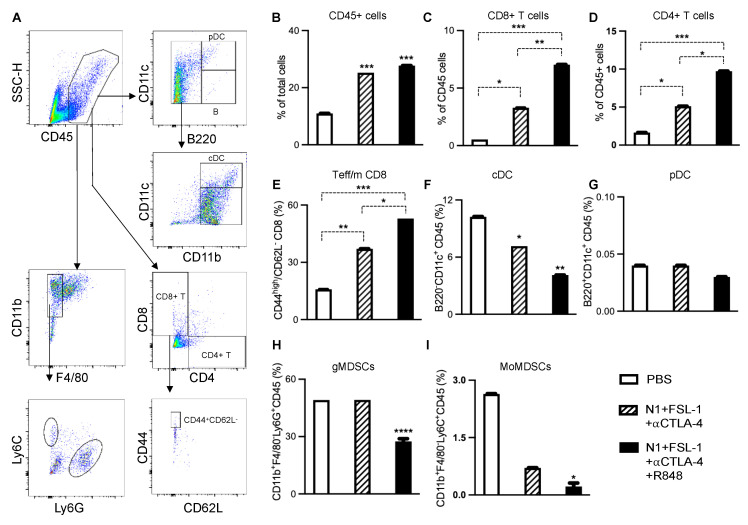
The effective combined immunotherapeutic regimens enhanced the immune activation in 4T1 mammary carcinoma model. Flow data were analyzed as in (**A**). Quantification of flow cytometry data showing elevated tumor infiltration of CD45+ leucocytes (**B**), CD8+ T cells (**C**), CD4+ T cells (**D**), and effector/memory CD8+ T cells (CD44highCD62L- CD8+ T cells (**E**), as well as reduced levels of infiltration of conventional dendritic cells (cDC; CD45+CD11c+B220- cells (**F**), granulocytic MDSCs (gMDSCs, CD45+CD11b+Ly6GhighLy6Clowcells (**H**), and monocytic MDSCs (Mo-MDSCs, CD45+CD11b+Ly6Glow Ly6Chigh cells (**I**). The level of plasmacytoid DC (pDC; CD45+CD11c+B220+ cells) in the tumor did not change in response to the combined immunotherapeutic regimens (**G**). Data are shown as mean ± SEM (n = 5). * *p* < 0.05; ** *p* < 0.01; *** *p* < 0.001; and **** *p* < 0.0001.

To link the observed antitumor efficacy of the employed TheraVac regimens to induced changes in DC and infiltrating immune cell phenotype, we examined immune cell populations of 4T1 tumors subject to different treatment regimens next. In this series of in vivo studies, the 1st dose of treatment was administered on day 7 post-implantation, and treatment continued until after application of the 3rd dose. Forty-eight hours after the last treatment, tumors and draining lymph nodes were harvested for the preparation of single-cell suspension and immunostaining. The number and phenotype of tumor-infiltrating leucocytes, myeloid-derived suppressor cells (MDSCs), and CD8 T cells were analyzed using FlowJo (Figure 4A). Treatment with N1, FSL-1, and anti-CTLA4 (TheraVacM) moderately enhanced the infiltration of CD45+ leucocytes (Figure 4B), CD4+ T cells (Figure 4D), CD8+ T cells (Figure 4C), and effector/memory T cells (Figure 4E). Infiltration of conventional DCs (Figure 4F) was reduced in comparison to the control group. The fraction of plasmacytoid (Figure 4G) DC (pDC) did not change compared to the control group, whereas the number of conventional DCs (cDC) was reduced (Figure 4F). In contrast, the combination of N1, FSL-1, R848, and anti-CTLA4 (TheraVac^plus^) further increased tumor-infiltrating leucocytes, CD4+ T cells and CD8+ T cells, effector/memory T cells, and markedly lowered fractions of tumor-infiltrating cDCs and pDCs. Thus, the significant increase of infiltration of immune cells, particularly CD4+ and CD8+ T cells induced by TheraVacPlus, suggests added value with respect to immunotherapeutic efficacy of R848 in 4T1 breast cancer. Further analysis of CD8+ T cells revealed that the abundance of effector/memory CD8 (defined as CD44^high^CD62L-) was elevated significantly. Furthermore, TheraVac^plus^ significantly reduced the recruitment of CD45+CD11b+F4/80-Ly6G+ immune cells known as granulocytic MDSCs (gMDSCs; Figure 4H) and CD45+CD11b+F4/80-Ly6C+ immune cells known as monocytic MDSCs (mMDSCs; Figure 4I) in the tumors. As both gMDSCs and mMDSCs contribute to the development of immunosuppression and resistance to immunotherapy, the reduced levels of gMDSC and mMDSC subpopulations seem to support a direct effect of the TheraVacplus regimen towards improved antitumor activity [26].

### 3.4. Effect of Combination Immunotherapy Using N1, FSL-1, R848, and Anti-CTLA4 (TheraVacplus) on Immune Cells in dLNs of 4T1 Murine Breast Tumors

**Figure 5:**
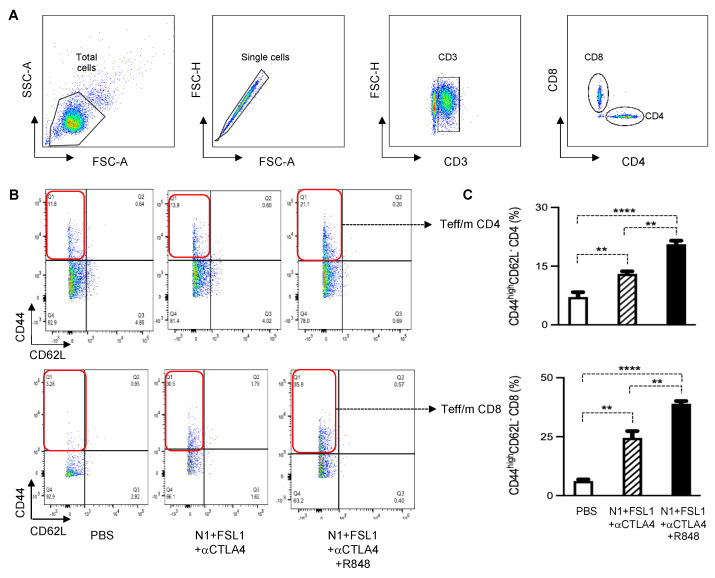
Treatment with the combination of N1, FSL-1, R848, and αCTLA-4 antibody elevated the levels on effector/memory CD4+ and CD8+ T cells in draining lymph nodes (dLNs). (**A**) Gating strategy. (**B**) The effector and memory (Teff/m) of CD4+ and CD8+ T cells of one representative mouse dLN. (**C**) The average (mean ± SEM, n = 5) levels of effector/memory CD4+ (CD4+CD44^high^CD62L-) and effector/memory CD8+ (CD8+CD44^high^CD62L-) T cells, respectively, in tumor dLNs. ** *p* < 0.01; and **** *p* < 0.0001.

**Figure 6:**
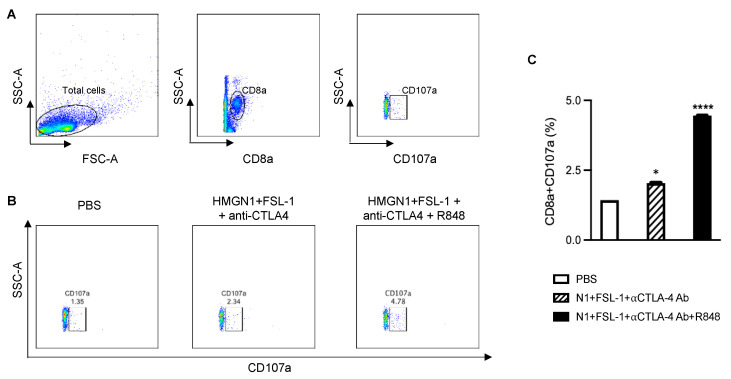
Treatment with the combination of N1, FSL-1, R848, and αCTLA4 antibody elevated the generation of 4T1-specific CTLs. (**A**) Flow cytometry gating strategy. (**B**) Representative plot of an individual mouse dLN. (**C**) The average (mean ± SEM, n = 5) levels of tumor-specific CTLs (CD107a+ CD8a+ T cells) in mouse tumor dLNs. * *p* < 0.05; and **** *p* < 0.0001.

To further gain insight into the mechanistic basis of combination therapy in the 4T1 breast cancer model, we harvested draining lymph nodes and stained with fluorescent dye-conjugated anti-CD3, anti-CD4, anti-CD8, anti-CD44, and anti-CD62L antibodies for flow cytometry. Gating strategies are shown in Figure 5A. The combination therapy TheraVac^M^ (N1, FSL-1, and anti-CTLA4) and TheraVac^plus^ (N1, FSL-1, anti-CTLA4, and R848) enhanced the effector/memory phenotype of CD4+ and CD8+ T cells in the dLNs as compared to the control (Figure 5B,C). Moreover, based on the coculture of dLNs with 4T1 tumor cells to determine the abundance of functional 4T1-specific CTLs, TheraVac^M^ and TheraVac^plus^ treatment also resulted in the generation of higher levels of functional 4T1-specific CD8+ T cells (CD8+CD107a+) in dLNs (Figure 6A–C) compared to the control/PBS treatment. Therefore, combination treatments promoted the generation of memory T cells and functional 4T1-specific CTLs in dLNs and infiltration of CD8 T cells in the tumors, observations consistent with the development of antitumor immunity.

**Figure 7:**
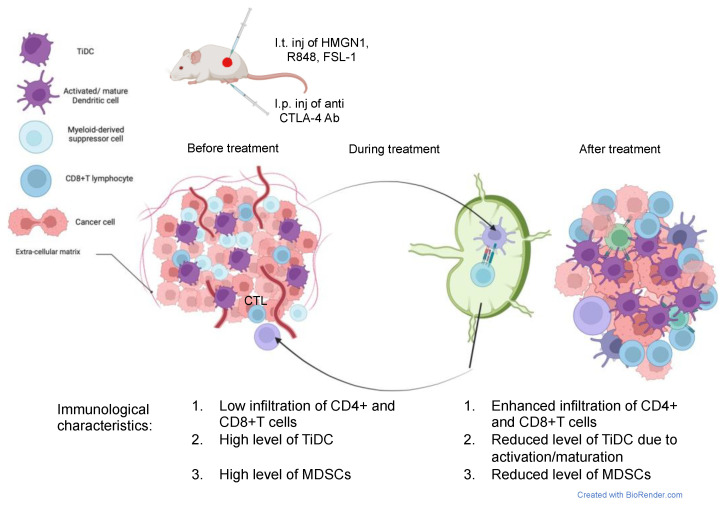
Schematic representation of the mechanism of action of combination therapy by HMGN1, FSL-1, anti CTLA-4 antibody, and R848.

In summary, combination immunotherapy using intratumoral administrations of N1, FSL-1, and R848 combined with CTLA4 ICB reprograms TiDCs, reduces MDSCs, and promotes an antitumor phenotype of tumor-infiltrating CD8+ T cells, overall converting immunologically cold 4T1 tumors into T-cell-inflamed lesions that are responsive to ICB (Figure 7).

## 4. Discussion

The highly malignant and poorly immunogenic 4T1 breast carcinoma is a challenging model for the evaluation of immune-related therapeutic modalities due to its high aggressiveness, immunogenic anergy, and poor response to any therapeutic strategy [27,28]. In the present work, we demonstrated the therapeutic efficacy and mechanistic basis of a multimodal immunotherapy approach consisting of the combination of TLR4 (N1), TLR2/6 (FSL-1), and TLR7/8 (R848) agonists administered intratumorally that can collaboratively promote maturation of DCs, and the systemically administered antagonizing CTLA4 antibody, which is termed TheraVacplus. Our data show that TheraVacplus exhibited significant antitumor, immunotherapeutic effects in 4T1 tumors, which were associated with tumor regression and, in a proportion of animals, tumor eradication. TheraVacplus treatment elevated levels of effector/memory CD4 and CD8 T cells in tumor tissue and draining lymph nodes and generated activated CD8+ T cells when cocultured with 4T1 cancer cells. Additionally, upon treatment with TheraVacplus, there was a concomitant reduction of gMDSCs in treated tumors. In view of the TheraVacplus favorable efficacy in immunologically “cold” 4T1 tumors, which is superior to conventional checkpoint inhibition or previous TheraVac regimens shown to be efficacious in other solid-organ models, these findings provide rationale for the translation of the TheraVacplus regimen for the treatment of human triple-negative breast carcinoma patients into the clinic.

Primary resistance to immune checkpoint blockade antibodies such as anti-PD1/PD-L1 or anti-CTLA4 is mainly associated with a lack of antitumor effector T cells including Th1 CD4 and CTLs, which correlates with low tumor mutational burden, inefficient presentation of tumor antigen(s), microbiota dysbiosis, and/or deficiency in IFNγ-mediated signaling in the tumors [29,30,31,32,33,34,35]. Mouse 4T1 mammary carcinomas resemble such “cold” tumors including human triple-negative breast carcinoma (TNBC) [36]. While patients with TNBC that expresses PD-L1 showed improved responses to a combination of pembrolizumab (anti-PD1) and paclitaxel, survival in the PD-L1high subgroup, however, was extended by a few months only [37]. TheraVac (combination of N1, R848, and an immune checkpoint blockade antibody) has previously been used to successfully treat other preclinical immunologically cold solid-organ tumors but failed to suppress the tumor growth of 4T1 breast carcinoma. 4T1 tumor growth reemerged under TheraVac treatment, implying the presence of additional immune-evasive molecular or cellular events. Thus, 4T1 breast carcinomas may possibly require inclusion of additional immuno-oncology targets to achieve tumor control. For example, it was recently shown that upregulation of inflammatory chemokine IL8 promoted tumor progression by stimulating the trafficking of MDSCs into TME, promotion of epithelial-to-mesenchymal transition of tumor cells, and by enhancing survival cancer stem-like cells in the tumor [38]. Thus, CXCR1/2 antagonism, either as monotherapy or in combination with immune checkpoint inhibitors, may be an effective immunotherapeutic combination. Prior studies showed that monotherapy with CXCR1/2 inhibitor SX682 or with anti-PD1 ICB alone had no significant effect on tumor growth or on the survival rate, but combination treatment reduced tumor growth and improved survival [39,40]. That depletion of MDSCs can sensitize tumors to ICB and improved outcome was recently elegantly shown by depleting the gMDSCs using anti-Ly6G mAb combined with anti-CTLA4, which achieved tumor rejection in 100% of mice with murine oral cancer, whereas anti-CTLA4 alone only led to tumor rejection in 45% of cases [41]. Similarly, combination treatment with SX682 and anti-PD1 antibody significantly reduced the growth of tumors compared to both untreated and anti-PD1-treated melanoma-bearing mice [42]. These findings of MDSC-targeted therapies improving the response to immunotherapy are in line with the improved tumor control of the TheraVac regimens coadministered with SX682 in our study. In our therapeutic strategy, we used several combinations along with SX682. The combination of TheraVacM and SX682 significantly reduced the growth of 4T1 tumors compared to control-treated mice and mice treated with TheraVacM alone. However, despite the encouraging tumor control, a curative effect was not reached by the combination of TheraVacM and SX682. Therefore, to achieve better therapeutic efficacy in 4T1 tumors, we added another TLR agonist R848 into TheraVacM to create TheraVacplus. TheraVacplus with or without SX682 further improved tumor control, achieving complete responses and tumor eradication in 20% of 4T1-bearing mice. The cured mice in both the TheraVacplus and TheraVacplus + SX682 groups remained tumor-free for up to 75 days. A previous study by Horn and coworkers in 4T1 tumor-bearing mice showed that the addition of SX682 to bintrafusp alfa (M7824; bifunctional fusion protein composed of the extracellular domain of the human TGF-β receptor II and combined with anti-PD-L1 moiety) synergistically delayed tumor growth and improved the response to therapy in the 4T1 model, although no tumor eradications were observed [43]. In this current study, SX682 played a role in delaying tumor growth further, but in the most effective group of treatment where curative effects were observed, the antitumor effect was, in large, driven by TheraVacplus.

Immunologically cold tumors like TNBC are significantly more challenging to treat due to their lack of T-cell infiltration and we need to devise alternative immunotherapy strategies. The reason behind the lower infiltration of T cells in 4T1 breast carcinoma is likely due to a lack of tumor-associated antigens and the inability of activated T cells to migrate towards the TME [44,45]. The data presented here imply that the treatment modalities using TheraVac, TheraVacM, and TheraVacM plus SX682 could not eliminate 4T1 tumors, whereas treatment with TheraVacplus resulted in further additional antitumor efficacy, which resulted in durable responses in a fraction of animals and a reduction of lung metastasis. Each of the components of TheraVacplus was essential in controlling tumor growth, as the removal of either R848 or FSL-1 resulted in diminished effectiveness of the combination. These findings attest to earlier in vitro findings of enhanced DC activation in the triple combination compared to N1 combined with FSL-1 alone, and that, after in vivo administration of the immunostimulants, synergistic mechanism within the combination TheraVac regimen remained preserved. In this regard, it remains to be determined whether consecutive administration compared to the current simultaneous administration might improve efficacy further, as initial activation of one mechanism might increase sensitization to the other components. For example, using the MMTV-PyMT mammary cancer model, Messenheimer and colleagues have shown in an elegant study that timing of the PD-1 blockade is critical to effective combination immunotherapy with anti-OX40 [46].

One of the concerns of combination immunotherapy is the immune-related adverse events (irAEs) due to systemic, off-tumor immune activation [47]. It is now well known from more than a decade of clinical practice with ICB that irAEs not only can lead to dose interruptions and discontinuation of ICB treatment, but in the most severe forms can be a fatal complication. Despite the observed intratumoral immune activation, our therapeutic combination was well tolerated in the treated mice with no measured weight loss, hair loss, and no behavioral change. Translationally, the intratumoral mode of administration, while possibly limiting the risks of systemic toxicities and successfully employed for earlier cytokine therapy trials, has been cited as a hurdle for acceptance into clinical practice. However, there is an increasing recognition of the advantages of the i.t. approach using the tumor as “its own vaccine” with an increasing number of trials testing novel immuno-oncology combinations which, due to systemic toxicities, the inability to formulate IO agents, or lack of target identification, cannot be given systemically [48]. These advantages apply to the TheraVac^M^ and TheraVac^Plus^ combinatorial approach, which can successfully treat 4T1 tumors without the need of identification, or administration of exogenous tumor-associated antigens. On the other hand, despite the presence of an increased amount of activated T cells, and increased numbers of CD8+ T cells in the N1/FSL-1/R848/anti-CTLA4 Ab group, the treatment doses or schedules documented here did not result in 100% cures, which suggests the presence or activation of a redundant immune-evasive mechanism. Furthermore, the intratumoral route of administration of N1, FSL-1, and R848 may exclude patients with either small or deep-seated, internal lesions which cannot be safely accessed by interventional radiology. Hence, we are currently investigating other formulation strategies suitable for the delivery of TheraVac^M^ or TheraVac^Plus^ to the tumor tissues upon systemic administration.

In summary, this work demonstrated the improved therapeutic efficacy achieving durable complete responses in the immunologically cold 4T1 murine breast cancer model of a novel combination immuno-oncology regimen comprising of the triple combination of N1, FSL-1, and R848 combined with CTLA4 ICB. The therapeutic approach (TheraVacplus) altered the 4T1 tumor immune landscape, promoting an antitumor effector phenotype and lessening immunosuppression, reducing tumor burden and lung metastasis in the 4T1 breast carcinoma model. Our study provides rationale for the clinical translation of TheraVacplus combination therapy as a novel treatment for highly aggressive and metastatic triple-negative breast carcinoma.

## 5. Conclusions

TNBC is the most aggressive type of breast cancer. Despite the availability of immune checkpoint blockade therapy, patients with immunologically cold TNBC respond poorly. Therefore, there is no effective therapy available for TNBC, and it is urgently needed to develop novel effective therapeutic approaches for treating TNBC. Utilizing the 4T1 murine model of TNBC, we tested the therapeutic effects of various combinations consisting of an immune-activating limb (HMGN1, FSL-1, and/or R848) and an immunosuppression-alleviating limb (ICB blockading antibody αCTLA4 and/or CXCR1/2 inhibitor SX682). 4T1-bearing mice responded with significant tumor regression and tumor elimination to our therapeutic combination regimen consisting of intratumoral delivery of high-mobility group nucleosome binding protein 1 (HMGN1), TLR2/6 ligand fibroblast-stimulating lipopeptide (FSL-1), TLR7/8 agonist (R848/resiquimod), and CTLA-4 blockade (termed TheraVac^Plus^). Additionally, treatment with TheraVac^Plus^ increased the number of infiltrating CD4+ and CD8+ effector/memory T cells in both tumors and draining lymph nodes and triggered the generation of 4T1-specific cytotoxic T lymphocytes (CTLs) in the draining lymph nodes. Thus, a potentially curative immunotherapeutic regimen for TNBC has been developed, which does not rely on the administration of chemotherapy, radiation, or exogenous tumor-associated antigen(s).

## Data Availability

This study does not involve usage of any mega dataset.

## Supplementary Materials

The following supporting information can be downloaded at: https://www.mdpi.com/article/10.3390/cancers15082366/s1, Figure S1: Gating strategy for analyzing MoDC expression of CD80, CD83, CD86, and HLA-DR; Figure S2: Synergistic activation of mouse bone marrow derived dendritic cells (BMDC) by HMGN1, R848, and FSL-1.
