# Supplementary material for: Regression and Eradication of Triple-Negative Breast Carcinoma in 4T1 Mouse Model by Combination Immunotherapies
Source: Cancers (Basel). 2023 Apr 19;15(8):2366. doi: 10.3390/cancers15082366 (PMC10136945; doi:10.3390/cancers15082366)
Supplement: Supplementary file 1 [file cancers-15-02366-s001.zip › cancers-2259820-supplementary.pdf]

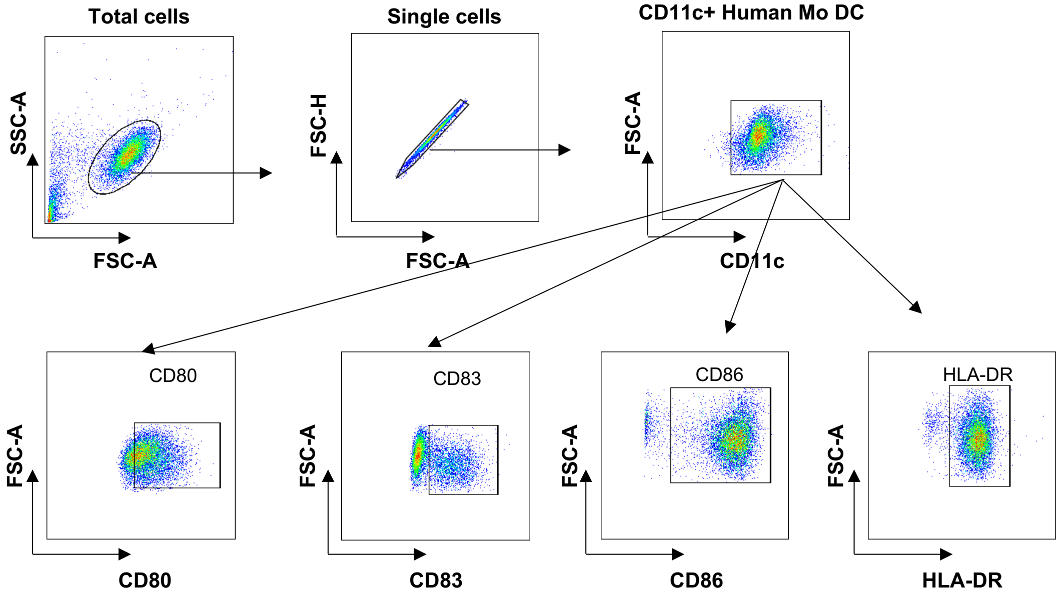

Supplementary Figure S1. Gating strategy for analyzing MoDC expression of CD80, CD83, CD86, and HLA-DR.

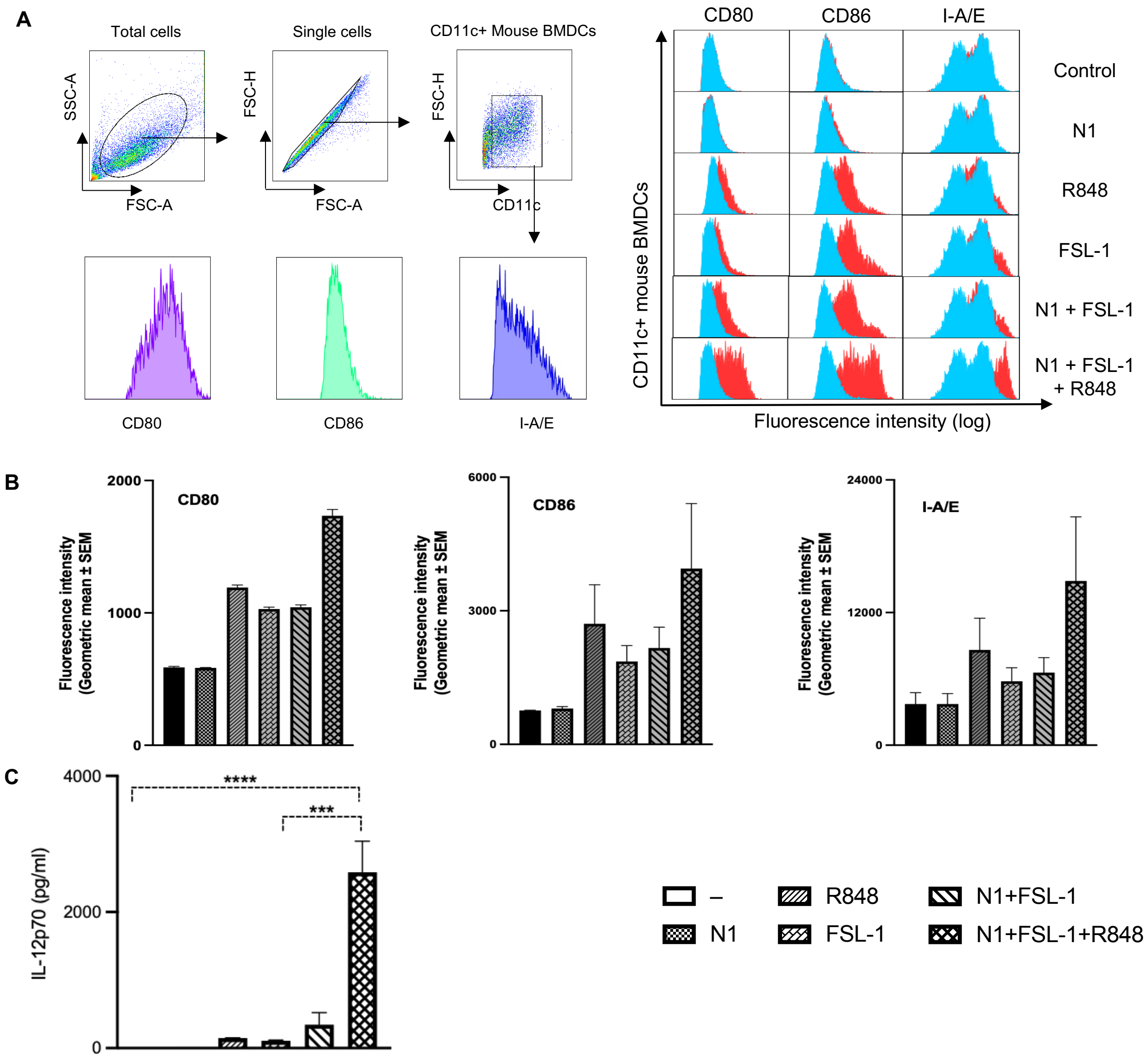

Supplementary Figure S2. Synergistic activation of mouse bone marrow derived dendritic cells (BMDC) by HMGN1, R848, and FSL-1. (A) Gating strategy and overlay histograms of BMDCs showing upregulation of CD80, CD86, and I-A/E in response to various treatments. (B) Average MFI (geometric mean ± SEM) of CD80, CD86, and I-A/E expression of three separate experiments. (C) IL-12 production in (pg/mL) in supernatants of BMDC cultures in response to indicated treatments (mean ± SEM, n=3).
